# Association between Serum Uric Acid Level and Metabolic Syndrome and Its Sex Difference in a Chinese Community Elderly Population

**DOI:** 10.1155/2014/754678

**Published:** 2014-07-23

**Authors:** Miao Liu, Yao He, Bin Jiang, Lei Wu, Shanshan Yang, Yiyan Wang, Xiaoying Li

**Affiliations:** ^1^Institute of Geriatrics, Chinese PLA General Hospital, 28 Fuxing Road, Beijing 100853, China; ^2^Beijing Key Laboratory of Aging and Geriatrics, Chinese PLA General Hospital, 28 Fuxing Road, Beijing 100853, China; ^3^State Key Laboratory of Kidney Disease, Chinese PLA General Hospital, 28 Fuxing Road, Beijing 100853, China; ^4^Department of Chinese Traditional Medicine and Acupuncture, Chinese PLA General Hospital, 28 Fuxing Road, Beijing 100853, China; ^5^Department of Geriatric Cardiology, Chinese PLA General Hospital, 28 Fuxing Road, Beijing 100853, China

## Abstract

*Objective*. This study aimed to evaluate the association between serum uric acid (SUA) levels within a normal to high range and the risk of metabolic syndrome (MetS) among community elderly and explore the sex difference. *Design and Methods*. A cross-sectional study was conducted in a representative urban area of Beijing between 2009 and 2010. A two-stage stratified clustering sampling method was used and 2102 elderly participants were included. *Results*. The prevalence of hyperuricemia and MetS was 16.7% and 59.1%, respectively. There was a strong association between hyperuricemia and four components of MetS in women and three components in men. Multiple logistic regression analysis showed ORs of hyperuricemia for MetS were 1.67 (95% CI: 1.11–2.50) in men and 2.73 (95% CI: 1.81–4.11) in women. Even in the normal range, the ORs for MetS increased gradually according to SUA levels. MetS component number also showed an increasing trend across SUA quartile in both sexes (*P* for trend < 0.01). *Conclusion*. This study suggests that higher SUA levels, even in the normal range, are positively associated with MetS among Chinese community elderly, and the association is stronger in women than men. Physicians should recognize MetS as a frequent comorbidity of hyperuricemia and take early action to prevent subsequent disease burden.

## 1. Introduction

Metabolic syndrome (MetS) is a complex of interrelated risk factors including obesity (particularly central adiposity), hyperglycemia, elevated blood pressure, hypertriglyceridemia, and decreased high density-lipoprotein cholesterol (HDL-C) [1]. Current available evidence suggests that MetS is associated with the development of diabetes, cardiovascular disease (CVD), and kidney diseases and is also of increased risk for mortality of CVD and all causes [2–6]. Also, population-based studies have shown that MetS is quite common, affecting about 13.7% of the middle-aged [7] and 50% of the elderly [8] in China. And its prevalence is increasing with dramatic socioeconomic change and a succession of increased unhealthy lifestyles [7].

Serum uric acid (SUA) is the end product of urine metabolism in human, and excess accumulation can lead to various diseases [9]. The relationship between SUA levels and cardiovascular conditions has been studied by a number of researches since 1960s [10–13]. However, the conclusion remains controversial, and SUA levels within a normal to high range have not been fully studied in association with MetS. In addition, most studies are primarily about adults and institutionalized people. There are limited studies focused on elderly population, which have higher prevalence of hyperuricemia and MetS and are of higher risk to develop CVD events. Data are needed to confirm the association of SUA levels with MetS and its individual components in community elderly population.

Moreover, a rapidly increasing trend of MetS prevalence has been reported in the Chinese elderly in the past decade, with an absolute change of 7.7% [14]. Therefore, it is important and urgent to identify the participants who have high risk of MetS and then to prevent subsequent related disease burden. Our study aims to examine the association of SUA levels and risk of MetS in an urban community elderly population of Beijing, China.

## 2. Materials and Methods

### 2.1. Methods

This was a population-based cross-sectional survey conducted in Wanshoulu Community of Haidian district, a metropolitan area representative of the geographic and economic characteristics in Beijing, China. The eligible subjects were individuals aged ≥60 years who had lived in the local district for at least one year. Sampling and research methods were reported elsewhere [14]. In brief, a two-stage stratified clustering sampling method was used. First, 5 residential communities were selected randomly from the total 36 residential communities. Second, all eligible households were chosen from the selected 5 communities. Between September 2009 and June 2010, a total of 2162 residents aged 60–95 years were selected and invited for screening. 2102 residents (848 men, 1,254 women) completed the survey. These participants accounted for about 10% of total elderly residents in the Wanshoulu community.

Each participant was interviewed and completed a standardized questionnaire including demographic factors, medical history, family history of CVD, and lifestyles. The height, weight, waist, and blood pressure were measured according to standardized protocol. Physical examinations and face-to-face interviews were carried out by specially trained nurses and physicians. Height was measured in meters (without shoes), and weight was measured in kilograms (with heavy clothing removed and 1 kg deducted for remaining garments). Body mass index (BMI) was calculated as weight in kilograms divided by the square of height in meters. Waist circumference on standing participants was measured midway between the lower rib margin and iliac crest. Two blood pressure recordings were obtained from the right arm of participants in a sitting position after 30 minutes of rest. If the difference between the first and second measurement was more than 5 mmHg, then repeated measurements were performed. The average of last two measurements was used. Overnight fasting blood specimens were obtained for tests of serum lipids, glucose, and SUA level. Samples were sent to the Central Certified Laboratory of Chinese PLA General Hospital in less than 30 minutes.

### 2.2. Definition of Hyperuricemia

Participants were diagnosed with hyperuricemia if their SUA level was ≥7.0 mg/dL (417 mmol/L) in men or ≥6.0 mg/dL (357 mmol/L) in women. SUA levels of participants without hyperuricemia were categorized into 4 levels using the quartiles (P_25_, P_50_, and P_75_) as cut-off values. In this study, sex-specific SUA quartiles for participants without hyperuricemia were as follows: Q1: ≤4.71 mg/dL (*n* = 176), Q2: 4.72–5.43 mg/dL (*n* = 176), Q3: 5.44–6.48 mg/dL (*n* = 175), Q4: ≥6.49 mg/dL (*n* = 173) in men; Q1: ≤3.90 mg/dL (*n* = 267), Q2: 3.91 mg/dL–4.54 mg/dL (*n* = 263), Q3: 4.55 mg/dL–5.10 mg/dL (*n* = 260), Q4: ≥5.11 mg/dL (*n* = 260) in women. All the four groups were under normal range of SUA level.

### 2.3. Definition of MetS

MetS was defined according to the 2009 harmonizing definition set by a joint statement of the International Diabetes Federation Task Force on Epidemiology and Prevention; National Heart, Lung, and Blood Institute; American Heart Association; Word Heart Federation; International Atherosclerosis Society; and International Association for the Study of Obesity, as the presence of three or more of the following five criteria: (1) central obesity: waist circumference (WC) ≥ 90 cm in Asian men and ≥80 cm in Asian women; (2) hypertriglyceridemia: fasting plasma triglycerides (TG) ≥1.7 mmol/L; drug treatment for elevated triglycerides is an alternate indicator; (3) decreased HDL-C: fasting HDL-C <1.0 mmol/L in men and <1.3 mmol/L in women; drug treatment for reduced HDL-C is an alternate indicator; (4) elevated blood pressure: systolic blood pressure (SBP) ≥ 130 mmHg and/or diastolic blood pressure (DBP) ≥ 85 mmHg; antihypertensive drug treatment in a patient with a history of hypertension is an alternate indicator; (5) hyperglycemia: fasting glucose level of ≥5.6 mmol/L (≥100 mg/dL); drug treatment of elevated glucose is an alternate indicator [1]. This harmonizing definition is the same with the modified NECP ATP III definition [15].

### 2.4. Statistical Analysis

Data were double entered using Epidata 3.1. All analyses were conducted using SPSS for windows (19.0, no. of serial: 5076595). Reported probabilities were two-sided; all tests were set at the 0.05 level of statistical significance.

Descriptive data were expressed as $\overset{-}{x}\pm s$ for continuous variables and *n*(%) for categorical variables unless otherwise specified.* t*-test and Chi-square test were used to examine differences in continuous and categorical variables. The relationship between SUA levels and other variables was assessed using the partial correlation coefficients. The trend test was used to determine MetS prevalence and its component number according to SUA quartiles. Finally, multivariable logistic regression was used to estimate the association between sex-specific SUA level and MetS prevalence. We calculated the odds ratio (OR) and 95% confidence intervals (CIs) of SUA for MetS and its five components.

### 2.5. Ethical Considerations

Ethical approval was obtained from the Ethics Committees of Chinese PLA General Hospital (EC0411-2001). All eligible participants had given their written informed consent.

## 3. Results

A total of 2,102 participants completed the survey, with 848 (40.3%) men and 1254 (59.7%) women. The mean age was 71.2 ± 6.6 (60~95 yrs); the older elderly (aged ≥ 80 yrs) accounted for 9.2%. The baseline characteristics of participants according to sex and hyperuricemia are presented in Table 1.

The prevalence of MetS and hyperuricemia in this community's elderly was 59.1%, and 16.7%, respectively. The percentage of participants who had both MetS and hyperuricemia was 12.4%; men were lower than women but with no statistical difference (11.0% versus 13.4%, *P* = 0.097).

### 3.1. Clinical Features of Participants with and without Hyperuricemia

Table 1 shows the demographic characteristics and anthropometric measurements of the 2102 participants. The mean SUA level was 5.3 ± 1.4 mg/dL (range, 1.4–11.3 mg/dL). In men, participants with hyperuricemia had older age, greater WC, higher BMI, higher TG, and lower HDL-C levels. However, there were no significant differences in blood pressure and FPG. In women, besides these differences, we also observed higher levels of 2-hour postprandial blood glucose (2hPG) in participants with hyperuricemia than those without. The percentage of participants who were married, better educated, currently smoking and drinking did not differ with hyperuricemia.

### 3.2. Correlation of SUA Level and Clinical Characteristics of Study Participants

Table 2 shows the correlation coefficients between SUA level and other clinical characteristics of the participants. SUA level was positively correlated with age, WC, BMI, TG, and 2hPG and negatively correlated with HDL-C in both sexes. In men SUA was also positively correlated with LDL-C and negatively correlated with FPG. In women, SUA was positively correlated with 2hPG and not correlated with TC, LDL-C.

### 3.3. Age and Sex-Specific Prevalence of Hyperuricemia in the Participants

**Figure 1:**
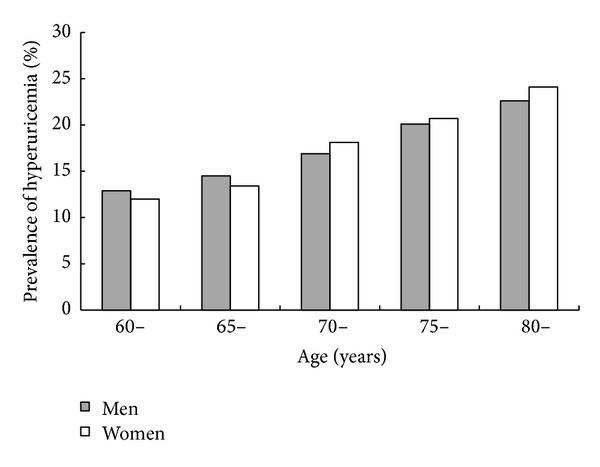
Age and sex-specific prevalence of hyperuricemia.

Figure 1 describes the prevalence of hyperuricemia by age and by sex. The prevalence showed an increasing trend with different age group (*P* for trend <0.001). Men had a higher prevalence in the younger elderly, but in the participants aged more than 70s, women had a higher prevalence.

### 3.4. Prevalence of MetS and Its Individual Components for Different Serum Acid Levels

The prevalence of MetS was greater in participants with hyperuricemia than those without in both sexes. In men, three individual components (excluding elevated blood pressure and hyperglycemia), and, in women, four individual components (excluding elevated blood pressure) showed significant difference in participants with and without hyperuricemia (*P* < 0.05). The number of MetS components also showed the same trend (Table 3).

Table 4 showed the prevalence of MetS and its five components for those without hyperuricemia. The prevalence of MetS increased from 39.2% and 50.6% to 55.5% and 70.4%, respectively, in men and women (*P* for trend <0.05 in both sexes) for those with SUA level under normal range. The number of MetS components also increased gradually with increasing SUA quartiles. In men, three of the individual components (excluding elevated blood pressure and hyperglycemia) and, in women, all five components showed increasing trends according to SUA quartiles (*P* for trend <0.05).

### 3.5. ORs of SUA and MetS

Table 5 showed the ORs of SUA for MetS and its individual components. Three types of SUA level were used, including hyperuricemia as a dichotomous variable, SUA level as a continuous variable, and quartiles as an ordinal categorical variable. The table showed that, after adjusted age, education, marital status, current smoking, current drinking, physical activity, family history, medications for hypertension, diabetes, and hyperlipidemia, participants with hyperuricemia or higher SUA level were at significantly elevated ORs for MetS and most of its five individual components.

There were sex differences of the ORs for MetS. Women had higher ORs of SUA level for MetS than men. The ORs for elevated blood pressure and hyperglycemia were not statistically significant in men using all three forms of SUA, but in women the ORs for the two components were significant using SUA levels as continuous variable and quartiles.

We also ascertained the association of SUA level and MetS in the sensitivity analysis and the results were similar (Tables 6 and 7). When participants with kidney disease (*n* = 105, 5.0%) were excluded, the adjusted ORs of hyperuricemia for MetS were 1.64 (95 CI: 1.09–2.47) in men and 2.68 (95 CI: 1.78–4.03) in women (Table 6). Also, in Table 7, when the participants aged under 65 years were excluded (*n* = 391, 18.6%), the adjusted ORs of hyperuricemia for MetS were 1.96 (95% CI: 1.26–3.06) in men and 2.55 (95% CI: 1.63–3.99) in women.

## 4. Discussion

In this study, we evaluated the association of SUA level and MetS in a Chinese community elderly population. We found that higher SUA level, even within normal range, was associated with an increased prevalence of MetS even after adjusting for confounding factors. This association of MetS risk by SUA levels was more robust in women than in men.

MetS and CVD are rapidly growing threats to public health worldwide, especially in economically developing countries such as China. Just in the past decade, prevalence of MetS in China has increased about 8% [14]. Considering the huge elderly people in China and the strong association of MetS with the development of CVD and diabetes [5], identifying high-risk asymptomatic individuals for MetS is of critical importance and may lead to improvements in prevention and treatment of the subsequent CVD events and increased socioeconomic burden.

A number of studies have researched the association between SUA levels and MetS or its individual components, primarily in adults or institutionalized people. There is little information about the situation in the community elderly, a population with higher prevalence of hyperuricemia and MetS and higher risk of developing CVD events. In this study, we investigated the main components of MetS in the aging population and their potential association with SUA levels. In this representative sample of the Chinese urban elderly, we found a graded positive association between hyperuricemia and the prevalence of MetS. Even in the normal range, SUA level still has higher remarkable increased risk of MetS. And MetS component number was found to rise along with the increase of SUA levels too. This is in accordance with previous studies. Several studies reported that there are significant associations of hyperuricemia with visceral or central obesity, high BMI, TGs, and vascular endothelial dysfunction [16]. A cohort study from Taiwan reported there was remarkable increase of 67% and 21% in the risk of MetS according to different SUA levels [11]. Significantly positive gradient between incidence of MetS and uric acid levels was observed among middle-aged and older participants in the Aerobics Center Longitudinal Study [17]. Also there are studies which indicated the mechanism that decreasing uric acid level may prevent or reverse the course of MetS. One possible biological mechanism is related to insulin-stimulated endothelial nitric oxide synthesis, and hyperuricemia may induce endothelial cell dysfunction and contribute to the development of MetS [18].

The prevalence of hyperuricemia was 16.2% in this population, with 17.5% in men and 16.3% in women, but with no significant difference (*P* = 0.475). After we calculate the age-specific prevalence, we can see men had a higher prevalence in the younger elderly, while in the participants aged more than 70s, the prevalence of hyperuricemia in women was higher than men. This is contrary to previous studies focused on adults [19, 20]. A meta-analysis including 59 studies of China showed the pooled prevalence of hyperuricemia in adult men was 21.6% (95% CI: 18.9%–24.6%), but it was only 8.6% (95% CI: 8.2%–10.2%) in adult women [21]. Different from these studies, we focused on the elderly and the women have all hit menopause. There is evidence showing estrogens in women may promote more efficient renal clearance of urate and explaining a substantial portion in the SUA level [20, 22]. Gouty arthritis and cardiovascular complications are rarely observed in premenopausal women; however, the incidences of hyperuricemia and MetS increase dramatically after menopause [20]. Also there is evidence which showed that when postmenopausal women with hyperuricemia took hormone replacement therapy, the SUA level and prevalence of hyperuricemia reduced significantly, which suggested a protective effect of estrogen [23, 24].

The present study also provides evidence that elevated SUA level was more strongly associated with MetS in women than in men. Previous studies that performed sex-specific analyses also reported similar results. Sex is clearly an important factor in the relationship between hyperuricemia and MetS. Women with a higher SUA concentration had a higher incidence of hypertension, hypertriglyceridemia, and low HDL-C, as well as increased cardiovascular morbidity and mortality compared to that in men [25]. A cohort study in the Taiwanese showed the hazards ratio of SUA level for MetS is 1.67 and 2.38 in women but only 1.24 and 1.38 in men compared with the lowest tertile [11]. But the underlying mechanism of the sex-related difference is still unclear and needed future investigation.

This is a population-based cross-sectional study with strict training process and quality assurance programs. Wanshoulu district is a representative metropolitan area of the geographic and economic characteristics in Beijing, such as income levels, residential status, and lifestyle factors. The response rate was high and there was no statistically significant difference between participants who completed and those who did not complete the data. Consequently, the findings are more representative than the previous ones, which are mainly about institutionalized patients or health screeners, and potentially reduce the selection bias. Second, our study showed that higher SUA levels, even in the normal range, are strongly associated with the prevalence of MetS. Most studies have revealed the association between obvious hyperuricemia and MetS. The results of our study showed higher SUA levels, even not high enough to the criteria of hyperuricemia, are of elevated risk of MetS. Third, this study explored the sex difference between the association of SUA level and prevalence of MetS, and a stronger association was revealed in women.

There are several limitations in this study which need to be considered. First, the nature of cross-sectional study did not allow us to assess the temporal relationship. Further cohort study with follow-up data is needed to verify the results. Second, the study only included participants who were aged ≥60 years and therefore may not be generalized to young people. But on the other hand, this aged population has a higher prevalence of developing MetS and CVD and would bring on bigger disease burden, so as to give us a critical alert to pay more attention to uric acid in the elderly with MetS and take early prevention actions to prevent serious CVD events. Third, the sex difference in the association of SUA level and MetS risk needs future investigations to explore the underlying mechanisms. Also, we did not have the information whether the women included in the study were taking hormone replacement therapy, which would have impact on both SUA level and MetS prevalence.

## 5. Conclusions

In summary, the present study showed a strong association between SUA and prevalence of MetS in Chinese community elderly population living in urban Beijing, even in the normal range of SUA level. It also provides additional evidence that SUA is more closely associated with the risk of developing MetS in women than in men. Physicians should recognize MetS as a frequent comorbidity of hyperuricemia and take early action to prevent subsequent chronic disease and related potential socioeconomic burden in the Chinese elderly.

## Figures and Tables

**Table 1:** Characteristics of the participants with hyperuricemia and those without.

Characteristics	Men (*n* = 848)				Women (*n* = 1254)				Total (*n* = 2102)
	Hyperuricemia (*n* = 148)	No hyperuricemia (*n* = 700)	*P* value	Subtotal	Hyperuricemia (*n* = 204)	No hyperuricemia (*n* = 1050)	*P* value	Subtotal	
$\overset{-}{x}\pm s$									
Age (yrs)	73.5 ± 6.9	72.1 ± 6.8	0.028	72.3 ± 6.9	71.8 ± 6.0	70.1 ± 6.3	0.001	70.4 ± 6.2	71.2 ± 6.6
WC (cm)	94.0 ± 8.4	90.6 ± 8.4	<0.001	91.2 ± 8.5	90.3 ± 9.5	85.6 ± 8.6	<0.001	86.4 ± 8.9	88.3 ± 9.1
BMI (kg/m^2^)	25.7 ± 3.0	24.8 ± 3.1	0.002	24.9 ± 3.1	26.4 ± 3.8	24.8 ± 3.5	<0.001	25.0 ± 3.6	25.0 ± 3.4
SBP (mmHg)	136.8 ± 17.5	136.6 ± 18.0	0.927	136.7 ± 17.9	141.9 ± 20.4	140.2 ± 20.9	0.285	140.5 ± 20.8	139.0 ± 19.8
DBP (mmHg)	77.4 ± 9.4	78.6 ± 9.7	0.191	78.4 ± 9.7	75.9 ± 10.4	76.8 ± 9.9	0.273	76.6 ± 10.0	77.3 ± 9.9
TC (mmol/L)^a^	5.0 ± 1.1	4.9 ± 0.9	0.131	4.9 ± 0.9	5.5 ± 1.0	5.5 ± 1.0	0.550	5.5 ± 1.0	5.3 ± 1.0
TG (mmol/L)	1.8 ± 1.0	1.5 ± 0.8	0.001	1.5 ± 0.9	2.1 ± 1.0	1.7 ± 0.9	<0.001	1.8 ± 0.9	1.7 ± 0.9
HDL-C (mmol/L)	1.2 ± 0.3	1.3 ± 0.4	0.016	1.3 ± 0.4	1.4 ± 0.4	1.5 ± 0.4	<0.001	1.5 ± 0.4	1.4 ± 0.4
LDL-C (mmol/L)^b^	3.1 ± 0.9	3.0 ± 0.8	0.338	3.1 ± 0.8	3.4 ± 0.8	3.4 ± 0.9	0.506	3.4 ± 0.9	3.3 ± 0.8
FBG (mmol/L)	6.0 ± 1.3	6.1 ± 1.6	0.428	6.1 ± 1.5	6.1 ± 1.3	6.1 ± 1.8	0.704	6.1 ± 1.8	6.1 ± 1.7
2hPG (mmol/L)	8.1 ± 2.6	8.2 ± 3.0	0.707	8.2 ± 3.0	9.2 ± 3.3	8.2 ± 3.3	<0.001	8.4 ± 3.3	8.3 ± 3.2
SUA (mg/dL)	7.9 ± 0.8	5.4 ± 0.9	<0.001	5.8 ± 1.3	7.0 ± 1.0	4.5 ± 0.8	<0.001	4.9 ± 1.2	5.3 ± 1.4
*n* (%)									
Education (≥6 yrs)	124 (83.8)	576 (82.3)	0.663	700 (82.5)	142 (69.6)	680 (64.8)	0.183	822 (65.6)	1522 (72.4)
Married	131 (88.5)	650 (92.9)	0.075	781 (92.1)	157 (77.0)	836 (79.6)	0.392	993 (79.2)	1774 (84.4)
Current smoking	27 (18.2)	153 (21.9)	0.329	180 (21.2)	7 (3.4)	44 (4.2)	0.615	51 (4.1)	231 (11.1)
Current drinking	58 (39.2)	267 (38.1)	0.812	325 (38.3)	14 (6.9)	86 (8.2)	0.522	100 (8.0)	425 (20.2)
PA (≥0.5 h/day)	88 (59.5)	422 (60.3)	0.852	510 (60.1)	121 (59.3)	603 (57.4)	0.618	724 (57.7)	1234 (58.7)
Family history of CVD	84 (56.8)	453 (64.7)	0.068	537 (63.3)	122 (59.8)	639 (60.9)	0.778	761 (60.7)	1298 (61.8)
Medication for hypertension	76 (51.4)	278 (39.7)	0.009	354 (41.7)	125 (61.3)	493 (47.0)	<0.001	618 (49.3)	972 (46.2)
Medication for diabetes	23 (15.5)	119 (17.0)	0.666	142 (16.7)	39 (19.1)	180 (17.1)	0.497	219 (17.5)	361 (17.2)
Medication for hyperlipidemia	31 (20.9)	86 (12.3)	0.006	117 (13.8)	38 (18.6)	147 (14.0)	0.088	185 (14.8)	302 (14.4)
MetS	93 (62.8)	332 (47.4)	0.001	425 (50.1)	168 (82.4)	649 (61.8)	<0.001	817 (65.2)	1242 (59.1)

Data are $\overset{-}{x}\pm s$ for continuous values or *n* (%) for category values; ^a^TC: total cholesterol; ^b^LDL-C: low density-lipoprotein cholesterol.

**Table 2:** Correlation of SUA and clinical characteristics of the study participants.

Characteristics	Men (*n* = 848)		Women (*n* = 1254)		Total (*n* = 2102)	
	*r*	*P* value	*r*	*P* value	*r*	*P* value
Age (yrs)	0.101	0.003	0.097	0.001	0.140	<0.001
WC (cm)	0.204	<0.001	0.239	<0.001	0.290	<0.001
BMI (kg/m^2^)	0.155	<0.001	0.224	<0.001	0.181	<0.001
SBP (mmHg)	0.021	0.547	0.046	0.105	0.002	0.920
DBP (mmHg)	−0.047	0.174	0.013	0.657	0.017	0.434
TC (mmol/L)	0.101	0.003	0.001	0.973	0.051	0.020
TG (mmol/L)	0.196	<0.001	0.199	<0.001	0.140	<0.001
HDL-C (mmol/L)	−0.152	<0.001	−0.185	<0.001	−0.227	<0.001
LDL-C (mmol/L)	0.097	<0.001	0.008	0.779	0.022	0.316
FBG (mmol/L)	−0.099	0.004	−0.004	0.884	−0.039	0.077
2hPG (mmol/L)	−0.028	0.422	0.112	<0.001	0.045	0.037

Adjusted for medication for hypertension, diabetes, and hyperlipidemia.

**Table 3:** Prevalence of MetS and individual components according to hyperuricemia.

	Hyperuricemia	No hyperuricemia	*P* value	Total
Men (*n* = 848)				
Individual components				
Central obesity	112 (75.7)	383 (54.7)	<0.001	495 (58.4)
Hypertriglyceridemia	73 (49.3)	244 (34.9)	0.001	317 (37.4)
Low HDL-C	51 (34.5)	181 (25.9)	0.033	232 (27.4)
Elevated blood pressure	122 (82.4)	545 (77.9)	0.217	667 (78.7)
Hyperglycemia	86 (58.1)	378 (54.0)	0.362	464 (54.7)
Number of MetS components				
One or more	142 (95.9)	654 (93.4)	0.246	796 (93.9)
Two or more	127 (85.8)	516 (73.7)	0.002	643 (75.8)
Three or more (MetS)	93 (62.8)	332 (47.4)	0.001	425 (50.1)
Four or more	56 (37.8)	165 (23.6)	<0.001	221 (26.1)
Five	26 (17.6)	64 (9.1)	0.003	90 (10.6)
Women (*n* = 1254)				
Individual components				
Central obesity	176 (86.3)	810 (77.1)	0.004	986 (78.6)
Hypertriglyceridemia	135 (66.2)	478 (45.5)	<0.001	613 (48.9)
Low HDL-C	117 (57.4)	431 (41.0)	<0.001	548 (43.7)
Elevated blood pressure	170 (83.3)	854 (81.3)	0.499	1024 (81.7)
Hyperglycemia	128 (62.7)	503 (47.9)	<0.001	631 (50.3)
Number of MetS components				
One or more	203 (99.5)	1000 (95.2)	0.005	1203 (95.9)
Two or more	191 (93.6)	887 (84.5)	0.001	1078 (86.0)
Three or more (MetS)	168 (82.4)	649 (61.8)	<0.001	817 (65.2)
Four or more	117 (57.4)	387 (36.9)	<0.001	504 (40.2)
Five	47 (23.0)	153 (14.6)	0.003	200 (15.9)

**Table 4:** Prevalence of MetS and individual components according to SUA quartiles for participants without hyperuricemia.

	Men (*n* = 700)					Women (*n* = 1050)				
	Q1	Q2	Q3	Q4	*P* value^a^	Q1	Q2	Q3	Q4	*P* value^a^
Individual components										
Central obesity	47.2	51.7	59.4	60.7	0.020	69.3	76.8	77.7	85.0	<0.001
Hypertriglyceridemia	27.8	29.0	41.1	41.6	0.001	33.7	41.8	51.9	55.0	<0.001
Low HDL-C	19.9	21.6	30.6	31.4	0.004	32.2	35.0	45.8	51.5	<0.001
Elevated blood pressure	76.1	75.4	79.5	80.3	0.547	73.8	81.5	82.9	87.3	<0.001
Hyperglycemia	52.3	48.9	58.3	56.6	0.181	41.2	46.3	49.2	52.1	0.007
Number of MetS components										
One or more	91.5	92.6	94.8	94.9	0.089	90.3	95.8	96.9	98.1	<0.001
Two or more	70.5	70.5	76.6	77.5	0.069	72.3	87.1	87.7	91.2	<0.001
Three or more (MetS)	39.2	44.3	50.9	55.5	0.008	50.6	60.8	65.8	70.4	<0.001
Four or more	14.8	17.6	30.6	31.4	<0.001	27.7	33.5	39.6	46.9	<0.001
Five	6.3	6.8	11.6	13.0	0.041	9.4	11.4	16.2	21.5	<0.001

^a^ *P* for trend.

**Table 5:** ORs and 95% CI of MetS and individual components according to SUA level.

	SUA level (continuous)		Hyperuricemia (dichotomous)		SUA level (quartiles for those under normal range)				
	OR (95% CI)	*P* value	OR (95% CI)	*P* value	Q1	Q2 OR (95% CI)	Q3 OR (95% CI)	Q4 OR (95% CI)	*P* value^a^
Men									
MetS	1.21 (1.08–1.36)	0.001	1.67 (1.11–2.50)	0.014	1.00	0.97 (0.66–1.43)	1.32 (0.76–2.30)	1.52 (1.03–2.23)	0.007
Number of MetS components	1.24 (1.14–1.36)	<0.001	1.81 (1.30–2.51)	<0.001	1.00	0.87 (0.60–1.27)	1.49 (1.02–2.18)	1.66 (1.13–2.45)	<0.001
Individual component									
Central obesity	1.25 (1.12–1.39)	<0.001	2.34 (1.55–3.52)	<0.001	1.00	1.01 (0.69–1.48)	1.50 (0.85–2.64)	1.65 (1.12–2.43)	0.027
Hypertriglyceridemia	1.27 (1.12–1.44)	<0.001	1.72 (1.13–2.64)	0.012	1.00	1.53 (1.00–2.33)	1.84 (1.27–2.90)	1.92 (1.02–3.35)	0.017
Low HDL-C	1.20 (1.03–1.39)	0.019	1.17 (0.68–2.01)	0.565	1.00	1.43 (0.91–2.27)	1.57 (0.82–2.98)	1.87 (1.19–2.93)	0.045
Elevated blood pressure	1.01 (0.88–1.07)	0.879	1.01 (0.60–1.72)	0.970	1.00	0.94 (0.60–1.50)	0.98 (0.62–1.57)	1.00 (0.50–2.00)	0.996
Hyperglycemia	1.10 (0.98–1.23)	0.098	1.22 (0.83–178)	0.321	1.00	0.95 (0.65–1.40)	1.12 (0.81–1.74)	0.90 (0.52–1.58)	0.641
Women									
MetS	1.50 (1.32–1.69)	<0.001	2.73 (1.81–4.11)	<0.001	1.00	1.59 (1.15–2.19)	1.85 (1.34–2.55)	3.05 (1.86–5.02)	<0.001
Number of MetS components	1.39 (1.28–1.52)	<0.001	2.11 (1.59–2.80)	<0.001	1.00	1.98 (1.45–2.70)	2.29 (1.14–3.63)	2.87 (2.09–3.95)	<0.001
Individual components									
Central obesity	1.32 (1.16–1.50)	<0.001	1.83 (1.19–2.82)	0.006	1.00	1.37 (0.95–1.97)	1.96 (1.34–2.87)	2.13 (1.20–3.75)	0.002
Hypertriglyceridemia	1.35 (1.22–1.50)	<0.001	2.28 (1.62–3.20)	<0.001	1.00	1.41 (1.02–1.94)	1.91 (1.39–2.63)	2.43 (1.56–3.80)	<0.001
Low HDL-C	1.32 (1.19–1.47)	<0.001	1.90 (1.36–2.67)	<0.001	1.00	1.33 (0.96–1.85)	1.89 (1.37–2.61)	2.58 (1.65–4.04)	<0.001
Elevated blood pressure	1.15 (1.02–1.31)	0.032	1.07 (0.71–1.61)	0.740	1.00	1.56 (1.05–2.32)	2.05 (1.11–3.78)	2.11 (1.40–3.17)	0.002
Hyperglycemia	1.23 (1.11–1.36)	<0.001	1.85 (1.32–2.59)	<0.001	1.00	1.50 (1.09–2.06)	1.19 (0.87–1.63)	1.78 (1.15–2.78)	0.021

Adjusted for age, education, marital status, BMI, current smoking, current drinking, physical activity ≥ 0.5 h/day, family history of CVD, medications for hypertension, diabetes, and hyperlipidemia.

^a^ *P* for trend.

**Table 6:** ORs and 95% CI of MetS and individual components excluding participants with kidney diseases.

	SUA level (continuous)		hyperuricemia (dichotomous)		SUA level (quartiles for those under normal range)				
	OR (95% CI)	*P* value	OR (95% CI)	*P* value	Q1	Q2 OR (95% CI)	Q3 OR (95% CI)	Q4 OR (95% CI)	*P* value^a^
Male (*n* = 765)									
MetS	1.21 (1.07–1.35)	0.002	1.64 (1.09–2.47)	0.018	1.00	1.45 (0.90–2.34)	1.67 (1.05–2.75)	2.30 (1.42–3.71)	0.008
Number of MetS components	1.23 (1.12–1.37)	<0.001	1.79 (1.28–2.50)	<0.001	1.00	0.87 (0.59–1.15)	1.47 (1.00–2.16)	1.60 (1.08–2.36)	0.033
Individual component									
Central obesity	1.23 (1.11–1.38)	<0.001	2.27 (1.50–3.43)	<0.001	1.00	0.79 (0.51–1.21)	1.29 (0.83–2.00)	1.33 (0.86–2.06)	0.048
Hypertriglyceridemia	1.26 (1.11–1.43)	<0.001	1.68 (1.09–2.59)	0.018	1.00	1.01 (0.58–1.76)	1.65 (0.87–2.82)	2.03 (1.20–3.43)	0.014
Low HDL-C	1.20 (1.03–1.39)	0.020	1.17 (0.68–2.01)	0.564	1.00	1.18 (0.59–2.34)	1.67 (0.86–3.22)	2.23 (1.17–4.24)	0.042
Elevated blood pressure	1.00 (0.87–1.16)	0.996	1.01 (0.59–1.73)	0.970	1.00	0.87 (0.49–1.55)	0.93 (0.58–1.49)	1.05 (0.78–1.92)	0.823
Hyperglycemia	1.10 (0.98–1.23)	0.104	1.22 (0.82–1.81)	0.324	1.00	0.97 (0.58–1.52)	1.45 (0.92–2.35)	1.53 (0.96–2.45)	0.084
Female (*n* = 1232)									
MetS	1.50 (1.32–1.69)	<0.001	2.68 (1.78–4.03)	<0.001	1.00	1.84 (1.24–2.72)	2.12 (1.41–3.19)	2.49 (1.65–3.76)	<0.001
Number of MetS components	1.39 (1.28–1.51)	<0.001	2.09 (1.58–279)	<0.001	1.00	2.01 (1.15–2.75)	2.37 (1.78–3.25)	2.89 (2.10–3.97)	<0.001
Individual components									
Central obesity	1.32 (1.16–1.50)	<0.001	1.83 (1.19–2.12)	0.006	1.00	1.47 (0.99–2.19)	1.65 (1.10–2.47)	2.65 (158–3.80)	0.001
Hypertriglyceridemia	1.35 (1.21–1.50)	<0.001	2.25 (1.60–3.17)	<0.001	1.00	1.81 (1.20–2.71)	2.38 (1.58–3.58)	2.47 (1.64–3.73)	<0.001
Low HDL-C	1.32 (1.18–1.46)	<0.001	1.88 (1.34–2.64)	<0.001	1.00	1.41 (0.93–2.15)	1.94 (1.27–2.94)	2.31 (1.52–3.50)	<0.001
Elevated blood pressure	1.18 (1.03–1.36)	0.009	1.08 (0.72–1.61)	0.132	1.00	1.62 (0.99–2.66)	1.72 (1.05–2.82)	2.25 (1.32–3.84)	0.018
Hyperglycemia	1.22 (1.10–1.36)	<0.001	1.83 (1.30–2.57)	<0.001	1.00	1.54 (1.03–2.31)	1.59 (1.07–2.38)	1.97 (1.321–2.92)	0.009

Adjusted for age, education, marital status, BMI, current smoking, current drinking, physical activity ≥ 0.5 h/day, family history of CVD, medications for hypertension, diabetes, and hyperlipidemia.

^a^ *P* for trend.

**Table 7:** ORs and 95% CI of MetS and individual components for participants aged more than 65 years.

	SUA level (continuous)		Hyperuricemia (dichotomous)		SUA level (quartiles for those under normal range)				
	OR (95% CI)	*P* value	OR (95% CI)	*P* value	Q1	Q2 OR (95% CI)	Q3 OR (95% CI)	Q4 OR (95% CI)	*P* value^a^
Male (*n* = 716)									
MetS	1.25 (1.10–1.42)	0.001	1.96 (1.26–3.06)	0.003	1.00	1.35 (0.80–2.29)	1.73 (1.01–2.96)	2.06 (1.22–3.47)	0.043
Number of MetS components	1.25 (1.13–1.39)	<0.001	2.01 (1.41–2.88)	<0.001	1.00	0.83 (0.53–1.22)	1.49 (0.96–2.28)	1.49 (1.00–2.00)	<0.001
Individual component									
Central obesity	1.22 (1.08–1.37)	0.001	2.18 (1.40–3.38)	0.001	1.00	1.28 (0.80–2.05)	1.57 (0.58–2.52)	1.63 (1.01–2.64)	0.054
Hypertriglyceridemia	1.28 (1.11–1.46)	0.001	1.96 (1.23–3.13)	0.005	1.00	1.11 (0.60–2.06)	1.66 (0.91–3.05)	1.84 (1.02–3.33)	0.046
Low HDL-C	1.19 (1.01–1.40)	0.040	1.30 (0.72–2.35)	0.377	1.00	0.75 (0.36–1.58)	1.21 (0.61–2.40)	1.40 (0.70–2.77)	0.047
Elevated blood pressure	1.05 (0.90–1.24)	0.532	1.10 (0.60–2.00)	0.767	1.00	0.80 (0.42–1.52)	0.97 (0.49–1.89)	1.10 (0.64–1.96)	0.880
Hyperglycemia	1.11 (0.99–1.25)	0.086	1.37 (0.90–2.08)	0.138	1.00	1.60 (0.97–2.67)	1.90 (1.13–3.18)	1.14 (0.68–1.92)	0.086
Female (*n* = 995)									
MetS	1.40 (1.23–1.59)	<0.001	2.55 (1.63–3.99)	<0.001	1.00	0.67 (0.43–1.04)	1.12 (0.71–1.76)	1.34 (0.85–2.11)	0.024
Number of MetS components	1.35 (1.23–1.45)	<0.001	2.07 (2.05–2.82)	<0.001	1.00	1.83 (1.29–2.59)	2.10 (1.48–3.00)	2.47 (1.73–3.54)	<0.001
Individual components									
Central obesity	1.25 (1.09–1.44)	0.001	1.82 (1.13–2.94)	0.014	1.00	0.80 (0.51–1.27)	1.10 (0.68–1.78)	1.57 (0.95–2.62)	0.045
Hypertriglyceridemia	1.27 (1.13–1.42)	<0.001	2.37 (1.63–3.46)	<0.001	1.00	0.59 (0.38–0.92)	1.12 (0.75–1.78)	1.15 (0.73–1.72)	0.015
Low HDL-C	1.29 (1.15–1.45)	<0.001	1.84 (1.26–2.68)	0.002	1.00	0.87 (0.54–1.39)	1.53 (0.96–2.43)	1.78 (1.14–2.84)	0.005
Elevated blood pressure	1.17 (1.00–1.34)	0.046	1.08 (0.71–1.62)	0.730	1.00	0.60 (0.34–1.06)	1.00 (0.55–1.83)	1.42 (0.73–2.78)	0.051
Hyperglycemia	1.23 (1.10–1.38)	<0.001	1.92 (1.33–2.78)	0.001	1.00	1.46 (1.02–1.84)	1.74 (0.78–1.86)	2.12 (1.50–2.74)	0.018

Adjusted for age, education, marital status, BMI, current smoking, current drinking, physical activity ≥ 0.5 h/day, family history of CVD, medications for hypertension, diabetes, and hyperlipidemia.

^a^ *P* for trend.
